# Vascular biomarkers in the prevention of childhood cardiovascular risk: From concept to clinical implementation

**DOI:** 10.3389/fcvm.2022.935810

**Published:** 2022-08-22

**Authors:** Henner Hanssen

**Affiliations:** Department of Sport, Exercise and Health, Faculty of Medicine, University of Basel, Basel, Switzerland

**Keywords:** children and adolescents, cardiovascular risk, primary prevention, vascular aging, biomarkers

## Abstract

Vascular biomarkers allow for non-invasive assessment of vascular structure and function and have been shown to be surrogates for cardiovascular (CV) outcome in adults. They reflect the cumulative risk of a plethora of single CV risk factors, such as obesity and hypertension, on the arterial wall. The process of atherosclerosis oftentimes has its origin in childhood and tracks into adulthood. Obesity-related CV risk in childhood is a main determinant of manifest CV disease and adverse outcome in adulthood. To date, prevention strategies are directed toward the detection and reduction of CV disease in adulthood. This review updates and puts into perspective the potential use of vascular biomarkers in children. With reference to the concept of early vascular aging in adults, it elaborates on the role of vascular biomarkers for CV risk stratification in children. The concept of primordial vascular aging implies that young children be screened for vascular health, in an attempt to timely detect subclinical atherosclerosis and initiate treatment strategies to reverse vascular damage in a period of life with high probability for risk regression. The evidence for the validity of macro- and microvascular candidate biomarkers as screening tools of CV risk in children is reviewed, and limitations as well as remaining research gaps are highlighted. Furthermore, an overview on the effects of exercise treatment on vascular biomarkers is given. Vascular biomarkers susceptible to lifestyle or drug treatment have the potential to qualify as monitoring tools to guide clinicians. This review discusses evidence for vascular biomarkers to optimize screening of childhood CV risk from initial concepts to potential future clinical implementation in cardiovascular prevention.

## Introduction

Cardiovascular (CV) disease remains the leading cause of death worldwide with highest prevalence rates beyond the age of 60 years. However, CV disease risk factors can oftentimes be detected early in life. Epidemiological surveys of the past 20 years demonstrate an increase in the population-based prevalence of elevated blood pressure and hypertension in children and adolescents, which is related to the concomitant increase of overweight and obesity in childhood (1). Childhood obesity and hypertension have become a global health hazard with an increasing prevalence of unhealthy and sedentary lifestyle among children (2, 3). Childhood physical inactivity plays a key pathophysiological role in the development of obesity-related CV disease and seems to be an important link between childhood risk factors and incidence of atherosclerosis in adulthood (4). The definition of common CV risk factors in children, including hypertension, dyslipidemia and hyperglycemia, have previously been defined in international expert consensus statements and guidelines, including suggestions for age-related cut-off values (5–7). Targeting these risk factors as early as childhood has the potential not only to improve childhood well-being but also to reduce CV disease manifestation in adulthood, as obesity-related childhood CV risk has been shown to track into adulthood (8–10). Young overweight children are four times as likely to become overweight adults than children with normal weight (11). In a population of 2.3 million adolescents, it was shown that adolescent obesity was associated with a substantially increased CV mortality in middle age (10). Baker et al. found that higher childhood body mass index (BMI) increased the risk of coronary heart disease (CHD) in adulthood (9). The longitudinal Cardiovascular Risk in Young Finns Study showed that elevated blood pressure in childhood tracks through adulthood (12). Systolic blood pressure in late adolescence has been shown to be an independent predictor for CHD and cerebrovascular stroke in mid-adulthood (13, 14).

Childhood CV risk factors are associated with the development and severity of vascular dysfunction and subclinical as well as manifest atherosclerosis in children and adults (15, 16). Circulating biomarkers such as high serum glucose and lipids as well as inflammatory markers and systemic effects of high blood pressure act on the vasculature, thereby inducing target organ damage in the macro- and microcirculation. As proposed by Nilsson et al., vascular target organ damage, be it structural or functional, represents a cumulative measure of overall CV risk in the process of aging (17). It therefore appears necessary to shed light on the potential of vascular surrogates of childhood CV risk in an attempt to improve CV risk stratification early in life. This review and outlook focuses on the evidence for the potential use of vascular biomarkers as a diagnostic screening tool for the prevention of childhood CV risk and the development of manifest CV disease later in life. First, it is reviewed how use of non-invasive vascular biomarkers emerged from post-mortem examinations of vascular pathology in children to allow for population-based screening and quantification of vascular aging in childhood. The existing concept of adult early vascular aging (17) is being translated into concepts applying for children. The overview on vascular biomarkers for CV risk stratification has a focus on carotid intima-media thickness (cIMT), forearm flow-mediated dilatation (FMD), arterial stiffness as well as recent findings of retinal vessel diameters as an innovative microvascular diagnostic approach. Based on this review, a concept-driven perspective for potential clinical implementation as future outlook is developed based on previous suggestions for use of vascular biomarkers in adults (18). This work did not aim at reviewing standard procedures of vascular imaging (“how to”) or discussing the benefits and pitfalls of the diagnostic approaches at length.

## Vascular surrogates of childhood cardiovascular risk

### The “coming of” vascular biomarkers in children

It is known from post-mortem studies in children and youth who died from accidents, violence or suicide, that the development of atherosclerotic plaque starts in early childhood and that the degree of subclinical alterations is associated with the presence of classical CV risk factors (19). In the Bogalusa Heart Study in 2–15 years old children, the number of risk factors was associated with the size of intimal fatty streaks and fibrous plaques (19). The Bogalusa Heart Study, as well as the Pathobiological Determinants of Atherosclerosis in Youth (PDAY) study (20) and the Muscatine Study (21), dating back to 1975, were the first studies to identify the origin of atherosclerosis in childhood. In these studies, childhood obesity was identified as the main driver for the development of atherosclerotic disease (19, 21). The PDAY study found an association of BMI ≥ 30kg/m^2^ as well as glucose intolerance with advanced vascular lesions at young age (20). These studies were of utmost importance for the understanding of the clinical relevance of childhood CV risk factors and their link to vascular organ damage. With reference to the famous quote of the English physician Thomas Sydenham (1624–1689) on age and vascular health in mankind, one may rephrase “A child is as old as its arteries.”

In the last two decades, subclinical non-invasive markers have been developed and applied to estimate the process of atherosclerosis and vascular aging in children and adolescence. In clinical settings, systemic and circulating biomarkers such as microalbuminuria, markers of systemic inflammation and oxidative stress have been used to establish associations with vascular damage and endothelial dysfunction. The use of direct non-invasive image analysis of vascular structure and function, for instance by ultrasound or tonometric devices, has enabled to refine primary prevention screening programs in childhood and has initiated the application of vascular biomarkers as surrogates for childhood CV risk. Vascular biomarkers of the macro- and microcirculation allow for early detection of subclinical CV risk and have the potential to allow monitoring of vascular aging from childhood to adulthood. The concept of childhood vascular aging and application of vascular biomarkers in childhood are reviewed in the following section.

### Concept of childhood vascular aging

Vascular aging is a gradual process involving biochemical and cellular modulations in the circulation. The concept implies that age-related clinical or subclinical manifestations are associated with vascular alterations, which can be quantified by sensitive non-invasive vascular assessments. The question remains, which accessible part of the vascular bed best mirrors the process of aging and is most sensitive to therapeutic approaches. Most importantly, which vascular biomarker is feasible and valid for application in children? Biomarkers that quantify potentially reversible structural and functional changes of the vascular bed are at the center of attention for monitoring preventive strategies.

The concept of CV risk assessment over the life course and the need for early prevention strategies, with a focus on lifestyle behavior, gradually developed over decades and was based on evidence in older adults from prospective long-term studies such as the Framingham study (22). The term early vascular aging (EVA) as a common principle for subclinical disease manifestation has been proposed by Nilsson et al. in order to offer better clinical guidance for adults with increased CV risk (17). Tissue biomarkers have the advantage of being a cumulative measure of the damaging effects of CV risk factors on the arterial wall and endothelial function. EVA describes the pronounced effects of aging on the vascular tree (23). Originally, the term was used primarily with a focus on central arterial stiffness. However, the concept also applies for other vascular beds such as the microcirculation. A cross-talk between the microcirculation and the macrocirculation exists, promoting a vicious circle of increased peripheral vascular resistance, increased central arterial stiffness, causing increases in central and peripheral blood pressures and eventually manifestations of vascular target organ damage (24).

**FIGURE 1:**
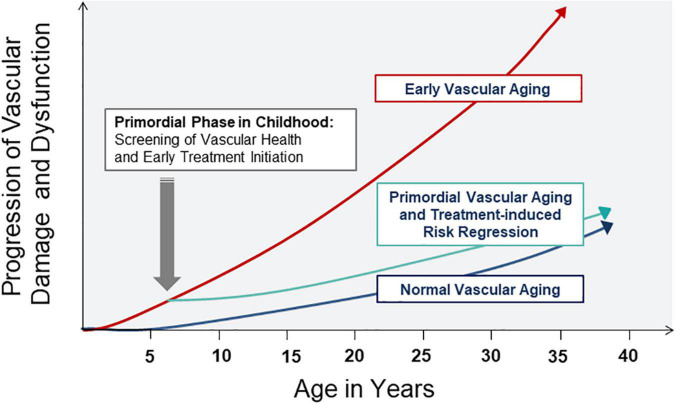
Concept of normal, primordial and early vascular aging across the lifespan. Screening in the primordial phase of vascular aging allows for timely detection of subclinical atherosclerosis and initiation of treatment strategies to reverse vascular damage and dysfunction in childhood and later in life.

Early vascular aging as a concept in adults can easily be transferred to CV risk stratification in young children. The concept of vascular aging in children refers to the primordial process of atherosclerosis, or indeed arteriolosclerosis, at young age. In the context of childhood CV prevention, the term “primordial” prevention has previously been discussed as a means for early detection of risk exposure and initiation of risk reduction strategies (25). Therefore, the term “primordial vascular aging” may be used as a term to best describe early vascular aging during childhood. The concept of primordial vascular aging in childhood is depicted in Figure 1, based on the initial concept in adults (17). Screening of vascular health should be performed early in life (primordial phase) in order to detect subclinical vascular alterations and initiate treatment strategies, such as dietary and exercise interventions, to start reversing the process of early vascular aging, at an age where regression of CV risk can be best achieved. Importantly, the reversibility of CV risk appears to be optimal in childhood rather than adulthood. For example, in a multi-center study of more than 6000 children and adolescence with a mean follow-up of 20 years, children who were successfully treated for high blood pressure and obesity during childhood had a comparable CV risk compared to lifelong healthy individuals (26, 27). However, if CV risk was prevalent and treated in adulthood, CV disease manifestation and complications such as myocardial infarction and stroke remained evident (26, 27). A much cited quote by the Italian statesman and philosopher Niccolo Machiavelli (1469–1527), originally meant as a comment on state affairs, puts the context of early prevention well into perspective: “…at the beginning a disease is easy to cure but difficult to diagnose; but as time passes, not having been treated or recognized at the onset, it becomes easy to diagnose but difficult to cure…”. An overview on the most common non-invasive vascular biomarkers and their clinical relevance in children is given in the following section.

## Vascular biomarkers of childhood cardiovascular risk

It appears eminent to define vascular biomarkers that are closely associated with obesity-related risk factors and hypertension in children (15, 19). The use of ultrasound-based diagnostic assessments of carotid intima-media thickness (cIMT) as a biomarker of large artery structure, and forearm flow-mediated dilatation (FMD) as a marker of large artery endothelial function are briefly reviewed in children. The focus is directed toward the use of pulse wave velocity (PWV) as a marker of large artery stiffness, and retinal vessel diameters as a marker of microvascular risk in children. Since new evidence for PWV and retinal vessel diameters has accumulated in recent years, these diagnostic approaches are reviewed in more detail. Moreover, potential effects of exercise interventions on vascular wall structure and function will be highlighted to indicate potential reversibility and sensitivity to treatment.

### Carotid intima media thickness

In adults, a vast amount of evidence exists for the use of cIMT and FMD as vascular biomarkers for risk stratification and prediction, both in the general population as well as in patients with CV disease (28, 29). However, according to the 2018 ESC/ESH Guidelines for the management of arterial hypertension in adults, it is currently not recommended to performed assessment of carotid imaging unless clinically indicated, for example in case of a previous transient ischemic attack (30). In childhood, less extensive data is available. Increased cIMT has been reported in children with obesity (31, 32) and high birth weight (32) as well as small size for gestational age (33). In the Bogalusa Heart Study (34) as well as the Cardiovascular Risk in Young Fins Study (35), obesity-related CV risk factors in childhood have been associated with higher cIMT in young adults. Hypertension and its severity has been shown to correlate with higher cIMT in children independent of obesity (35). The association of childhood blood pressure with cIMT has been confirmed in a systemic review, but with no recommendation for cut-off values (34). The Working Group on Cardiovascular Prevention of the Association for European Paediatric Cardiology (AEPC) has previously published a statement on practical guidelines for the measurement and interpretation of cIMT values in children (36). Several publications with suggestions for normative data in children are presented in the statement, with limitations in sample size and heterogeneity in the applied methods. It is concluded that comparison of different measurement approaches and standardized protocols are warranted (36). A general consensus for normative data in children is still lacking. In principle, percentiles are used as a statistical term to indicate deviation of the norm during childhood development and cIMT values above the 95^th^ percentile can be are considered pathological (37, 38).

### Flow-mediated dilation

Reduced FMD, as a marker of large artery endothelial function, has also been associated with childhood obesity (39, 40). However, even though FMD appeared to be feasible and reproducible in children aged 9-12 years, FMD was positively associated with BMI in the large-scale Avon Longitudinal Study of Parents and Children (ALSPAC) including over 6000 children aged 10–12 years (41). This unexpected finding remained unexplained, but may perhaps be attributed to the high variability of developmental status in this age-range in the proximity of puberty. As a further risk factor, low birth weight has been associated with reduced FMD (42, 43). Moreover, passive smoking has been shown to impair FMD in a dose-dependent manner (44). In addition, a recent meta-analysis demonstrated an impaired FMD in children with type 1 diabetes (45). Nonetheless, data on FMD in children remain limited and this may, at least in part, be due to the fact that sustained cuff dilatation for 5 min is not well tolerated in young children. With respect to normative data, it has been proposed that FMD during childhood does not alter (46). Indeed, it appears that age- and sex-specific data for FMD are not needed on pediatric populations between the age of 6–16 (47). Normal FMD in children has thus been suggested to lie in the range of 7.7–8.2% (47).

Both cIMT and FMD have been shown to be sensitive to lifestyle treatment in early life. In a pioneer study by Meyer et al., regular aerobic exercise for 6 months reduced cIMT and improved FMD in children with obesity (40). In two meta-analysis, exercise interventions have been shown to improve FMD (48) and reduce cIMT (49) in children with overweight and obesity. Both diet alone and diet combined with exercise have been shown to improve cIMT and FMD in overweight children, with sustained improvements after 1 year in the combined lifestyle group (50). The reversibility of cIMT in children is of particular note, as increased cIMT cannot be reversed by long-term exercise training in middle-aged men, but only its progression can be attenuated in individuals not taking statins (51).

### Arterial stiffness

Arterial stiffness is typically measured by use of tonometric devices as an estimate of aortic pulse wave velocity (PWV) using the carotid and femoral sites. In a subclinical stage of arteriosclerosis, the aorta and major arteries stiffen and steadily lose their elastic and distensible abilities (52). Increased arterial stiffness is a sign of grave structural and functional alterations of arterial wall integrity. The assessment of large artery stiffness has become a validated and recommended vascular biomarker as a surrogate end point for cardiovascular risk assessment in adults (53–56). An increase of PWV by 1 m/s has been shown to represent a risk increase of 15% in total CV and all-cause mortality in adults (53). Aerobic exercise training has been shown to have positive effects on arterial stiffness in congestive heart failure (57), end stage renal disease (58) and chronic obstructive pulmonary disease (COPD) (59). A recent meta-analysis of fourteen trials, including 472 pre- and hypertensive subjects, revealed that a reduction of arterial stiffness following aerobic exercise was dependent on a substantial reduction in systolic blood pressure and prolonged exercise duration (60). The European Society of Hypertension (ESH) as well as the European Society of Cardiology (ESC) have recommended central PWV as a tool for the assessment of subclinical target organ damage (61). Recent studies demonstrated that a PWV value of >10 m/s can already be considered as being a threshold to an abnormal, high-risk CV status (56, 62, 63). In adults, it has previously been suggested by the Reference Values for Arterial Stiffness‘ Collaboration to use values according to age groups rather than absolute cut off values (63).

Such recommendations and thresholds are still lacking for use across the age span of children. Suggestions for reference values in children have been put forward but have not achieved general consensus (64, 65). Further reference values have been suggested for tonometric (66) as well as oscillometric systems (67, 68) in cohorts of 1000 individuals and more throughout childhood until early adulthood.

An increasing number of studies demonstrate the association of arterial stiffness with multiple CV risk factors in childhood. Tounian et al. in 2001 were one of the first to show that severe obesity was associated with increased local arterial wall stiffness in children by use of ultrasound assessments of the common carotid artery (69). In the Cardiovascular Risk in Young Fins Study, childhood risk factors such as high LDL and low HDL cholesterol, higher fat mass and blood pressure were associated with local carotid stiffness in young adults (70). Using central PWV as an estimate of large artery stiffness, Sakuragi et al. in 2009 demonstrated that higher BMI and lower cardiorespiratory fitness were associated with higher PWV in prepubescent children (71). In a meta-analysis by Cote et al., childhood obesity was confirmed to be associated with higher PWV (72). In our more recent systematic review and meta-analysis, we found higher systolic and diastolic blood pressure and BMI to be associated with higher PWV and higher cardiorespiratory fitness to be associated with favorably lower PWV (73). In the EXAMIN YOUTH Follow-up study, we identified increased blood pressure and progression of microvascular dysfunction to be determinants of higher PWV after 4 years follow-up in prepubescent children (74). In this study, habitual vigorous physical activity had the potential to decelerate arterial stiffening over the 4- year time span. Interestingly, even high levels of parental physical activity, specifically maternal activity, has been associated with development of lower PWV in children (75). It has previously been argued that arterial stiffness in childhood may track into adulthood and can be considered part of the etiology of adult cardiac dysfunction (16). In turn, a recent meta-analysis also showed that elevated blood pressure in childhood was associated with higher PWV, carotid intima media thickness (cIMT), CV disease and overall mortality in adulthood (76). It has to be mentioned that the effects of childhood development on arterial stiffness in the proximity of puberty are a matter of ongoing debate. In the above mentioned ALSPAC study, greater childhood obesity was not associated with significantly higher arterial stiffness or endothelial dysfunction in children aged 10-12 years (77). It is concluded that early childhood may be a “window of opportunity” to detect and target vascular alterations in the young.

### Retinal vessel diameters

Retinal vessel analysis is an innovative non-invasive technique that allows examination of the retinal microcirculation (78). The interest in retinal vessels is based upon the fact that they are regulators of local cerebrovascular blood flow and can be analyzed very easily. Ninety percent of endothelial cells are found in the small vessels of the microcirculation, and it therefore appears plausible to use the microcirculation for early detection of endothelial dysfunction. In addition, retinal vessel analysis allows the investigation of alterations of venous structure and function in the microcirculation (79). In adults, large cohort studies have shown that narrower arteriolar and wider venular vessel diameters, resulting in a lower arteriolar to venular diameter ratio (AVR), are associated with increased risk and severity of hypertension (80–82), risk of stroke (83, 84) and a higher CV morbidity and mortality in older subjects (85, 86). In a meta-analysis of adult populations including more than 44 000 individuals, higher BMI was found to be associated with both narrower retinal arterioles and wider venules (87). The Blue Mountains Eye Study showed that normotensive persons with retinal arteriolar narrowing were more likely to develop hypertension and those with mild hypertension to progress to severe hypertension (88). In the Atherosclerosis Risk in Communities Study (ARIC), arteriolar narrowing and venular widening predicted incidence heart failure and adverse cardiac remodeling after 18 years of follow-up (89).

In children and adolescents, narrower retinal arteriolar diameters and wider retinal venular diameters have also been associated with CV risk factors (90, 91). A previous meta-analysis of our group including 11 studies revealed that children and adolescents with obesity or hypertension have narrower arteriolar vessel diameters compared to their lean and normotensive peers (92). An increasing BMI has been associated with increasing retinal venular diameters, and diameter changes in 8-year old children have been shown to increase the risk of higher BMI five years later (93). We have recently shown that arteriolar narrowing in childhood is predictive for the development of high blood pressure four years later (94). The Young Finns Study in turn gave first evidence that elevated blood pressure in childhood was associated with narrower retinal arteriolar diameters in mid-adulthood (95). Evidence for the association of physical activity and retinal vessel diameters as a vascular biomarker for CV disease in children has been accumulating in recent years. Gopinath et al. have shown an association between higher physical activity levels and wider retinal arteriolar diameters (96). Our group has previously found that physical inactivity was associated with wider venular diameters and a resulting lower AVR in young school children (97). Moreover, we have shown that higher cardiorespiratory fitness, as assessed by shuttle run stages, was associated with narrower retinal venular diameters and a favorably higher AVR in 6-8 years old children (98, 99). In the only longitudinal study available, we have shown that children with an improved cardiorespiratory fitness reduced their BMI and, thereby, developed wider arteriolar vessel diameters over four years (100). In children with elevated blood pressure at baseline, a reduction in sedentary behavior was associated with the development of wider arteriolar vessel diameters after 4 years of follow-up (100). In youth, an 8 weeks multimodal exercise program during school breaks was found to induce widening of retinal arteriolar diameters, which was associated with improvements in cognitive function in adolescence (101). In a cluster randomized school-based intervention program, lifestyle counseling and health education for 18 months has been shown to improve retinal microvascular heath in children (102). From our recent systematic review on exercise and retinal vessel diameters it became evident, that physical activity and exercise interventions have the potential to counteract microvascular remodeling in adults as well as children, thereby preventing development of small vessel disease across the lifespan (103). Given the predictive value for CV disease outcome in adults and risk factor development in children, retinal vessel diameters are a promising candidate microvascular biomarker for CV risk stratification across the lifespan. Normative data for retinal vessel diameters have recently been published for adults (104), however, such data for children across the age span are still warranted.

## From concept to potential clinical implementation

**FIGURE 2:**
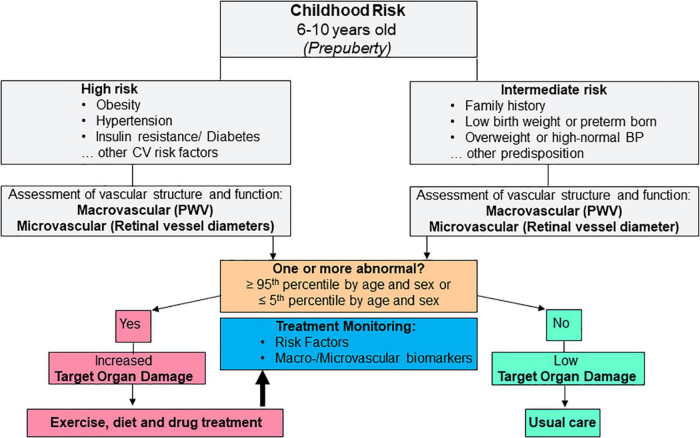
Example for potential future clinical implementation of vascular biomarkers as a means for cardiovascular risk stratification and treatment monitoring during childhood. CV, cardiovascular.

The concept of premature vascular aging, derived from the principle of EVA in adults, implies that screening of CV health is performed in a premature phase of vascular impairments in early childhood. Vascular biomarkers can be easily and non-invasively applied in children with the advantage of reflecting cumulative CV risk in a decisive target organ. The presented evidence suggests that the preventive approach is best applied in prepuberty, for example between the age of 6–10 years. Children need to be able to adhere to the instructions and comprehend the principle procedures. As aggravated childhood development in the proximity of puberty may mask subclinical vascular alterations, risk stratification should preferably be performed before puberty. Treatment of obesity-related CV risk in children is, first and foremost, based on exercise and dietary interventions. Exercise interventions, as shown in the previous chapter, are effective means to improve vascular health in children with the potential to reverse premature vascular aging and achieve considerable risk regression with reduction of risk trajectories into adulthood. Based on this principle concept, it is necessary to develop a practical algorithm for clinical implementation. Similar to a previous recommendation for CV risk stratification by use of vascular biomarkers in adults (18), children pre-identified as having intermediate (for example family history of CV disease, overweight or elevated blood pressure) or high (for example obesity, hypertension, diabetes) CV risk may benefit the most from vascular screening programs and subsequent lifestyle or drug treatment. According to this principle, an example for concept-driven potential clinical implementation is depicted in Figure 2. Prepubertal children with intermediate or high risk should undergo assessment of vascular structure and function. These should include more than a single vascular biomarker of the macro- and microcirculation, for example carotid-femoral PWV and retinal vessel diameters as two promising candidate biomarkers of two different vascular beds. If none of these two biomarkers is beyond the 95^th^ percentile (≤ 5^th^ percentile for retinal AVR) by age and sex, target organ damage is low and one can proceed with usual care. In case one or more biomarkers are beyond the 95^th^ percentile (≤ 5^th^ percentile for retinal AVR), increased target organ damage is evident and treatment needs to be initiated or intensified, for example by prescription of exercise or dietary interventions or drug treatment.

## Limitations and research gaps

The above stated possible cut-off values remain debatable and need to be established for each individual biomarker. In principle, the deviation from the norm in pediatrics is perhaps best defined as a cut-off point of below the 5^th^ percentile or above the 95^th^ percentile, especially when used as indication for initiation of pharmacological treatment. No study to date has investigated achievable treatment targets based on retinal vessel diameters or PWV as the primary end point in children. Until these data are available, the proposed concept for clinical decision making based on retinal vessel diameters and PWV remains hypothetical and helps identify research gaps rather than representing recommendations for clinical practice.

In adults, vascular biomarkers such as retinal vessel diameters but also inflammatory markers have been associated with the diagnosis and prognosis of cognitive impairments and even dementia (105, 106). Few studies have investigated the association of biomarkers of vascular health with cognitive function in children. Retinal vessel diameters, for example, have been found to independently explain a considerable proportion of variance in cognitive function in healthy children (107) and exercise-induced improvements in cognitive function have been shown to be accompanied by widening of retinal arterioles (101). More research is warranted to investigate the link between vascular health and brain function in children.

Treatment efficacy on CV risk reduction should be monitored not only by reassessment of risk factors but also by monitoring vascular health. This implies that vascular biomarkers not only have a high validity and reproducibility in children but are also sensitive to treatment. Moreover, normative data and standardized operating procedures in children are warranted. Even though there is good evidence for use of the presented vascular biomarkers, reliable normative data and consensus on standardized procedures are still lacking across the childhood age span. To this respect, few advances have been achieved in the last decade as these research gaps had already been identified in a scientific statement from the American Heart Association dating back to 2009 (108). The type and frequency of screening as well as the setting and target populations beyond prevalence of risk factors also remain a matter of debate. In order to pave the way for clinical implementation of such concepts, future research will have to meet these research gaps and address the need for prospective long-term follow-up studies to verify the benefit of childhood vascular screening programs to reduce childhood risk trajectories from childhood to adulthood, and reduce the burden of manifest CV disease later in life. The use of sophisticated algorithms of artificial intelligence in vascular signaling as well as new approaches in telemedicine may further support and accelerate clinical implementation.

## Conclusion

This review presents concept-driven evidence for the potential clinical implementation of vascular biomarkers in the prevention of CV risk and disease manifestation to reduce risk trajectories from childhood to adulthood. In addition to screening for common circulating risk factors, the use of non-invasive vascular biomarkers may proof to be of long-term clinical value to determine cumulative overall CV risk in children. The presented vascular biomarkers have been shown to be susceptible to lifestyle interventions as the primary treatment option for childhood CV risk reduction starting before puberty. Thus, they have the potential to qualify as monitoring tools and help guide individual treatment strategies. However, several limitations and research gaps remain which is why current guidelines do not currently support their routine clinical use. Recent findings for the use of retinal vessel diameters as microvascular biomarkers of CV risk in children perhaps resemble most promising developments in the field. The added value of implementing vascular biomarkers on top of usual care has yet to be shown in prospective long-term studies overarching childhood development into adulthood. Even though common CV risk factors have been shown to track into adulthood, future studies will have to investigate the extent at which remodeling of the vascular bed tracks into adulthood and is related to incidence manifest CV disease. Most importantly, standard operating procedures and normative data have to be defined for all age groups before concepts for clinical implementation can be evaluated.

## Author contributions

The author confirms being the sole contributor of this work and has approved it for publication.
